# US Public Support for Vaccine Donation to Poorer Countries in the 2009 H1N1 Pandemic

**DOI:** 10.1371/journal.pone.0033025

**Published:** 2012-03-06

**Authors:** Supriya Kumar, Sandra Crouse Quinn, Kevin H. Kim, Karen M. Hilyard

**Affiliations:** 1 Department of Epidemiology, Graduate School of Public Health, University of Pittsburgh, Pittsburgh, Pennsylvania, United States of America; 2 Department of Family Science, School of Public Health, University of Maryland, College Park, Maryland, United States of America; 3 School of Education, University of Pittsburgh, Pittsburgh, Pennsylvania, United States of America; 4 Department of Health Promotion and Behavior, College of Public Health, University of Georgia, Athens, Georgia, United States of America; UCL Institute of Child Health, University College London, United Kingdom

## Abstract

**Background:**

During the 2009 H1N1 pandemic, the global health community sought to make vaccine available “in developing nations in the same timeframe as developed nations.” However, richer nations placed advance orders with manufacturers, leaving poorer nations dependent on the quantity and timing of vaccine donations by manufacturers and rich nations. Knowledge of public support for timely donations could be important to policy makers during the next pandemic. We explored what the United States (US) public believes about vaccine donation by its country to poorer countries.

**Methods and Findings:**

We surveyed 2079 US adults between January 22^nd^ and February 1^st^ 2010 about their beliefs regarding vaccine donation to poorer countries. Income (p = 0.014), objective priority status (p = 0.005), nativity, party affiliation, and political ideology (p<0.001) were significantly related to views on the amount of vaccine to be donated. Though party affiliation and political ideology were related to willingness to donate vaccine (p<0.001), there was bipartisan support for *timely* donations of 10% of the US vaccine supply so that those “at risk in poorer countries can get the vaccine at the same time” as those at risk in the US.

**Conclusions:**

We suggest that the US and other developed nations would do well to bolster support with education and public discussion on this issue prior to an emerging pandemic when emotional reactions could potentially influence support for donation. We conclude that given our evidence for bipartisan support for timely donations, it may be necessary to design multiple arguments, from utilitarian to moral, to strengthen public and policy makers' support for donations.

## Introduction

The first few cases of 2009 H1N1 influenza were detected in Mexico and California in April 2009. Subsequently, the virus rapidly spread around the world and a pandemic was declared by the World Health Organization (WHO) on 11^th^ June 2009 [1]. With the advent of the pandemic, significant attention turned to the timely production of vaccine to prevent morbidity and mortality and the first vaccines became available in the United States (US) in October 2009 [2]. Attention also turned to ensuring equity in the global distribution of vaccine to countries unable to afford or finance vaccines themselves. Specifically, the WHO director-general called for “international solidarity to provide fair and equitable access to pandemic influenza vaccines for all countries” [1], [3]. The Bill & Melinda Gates Foundation proposed a set of ethical principles to “guide the global allocation of pandemic vaccines,” including “availability of vaccine in developing nations in the same timeframe as developed nations” [4]. In practice, equitable access to vaccine would mean that countries concurrently (or in the same timeframe) receive a proportionate share of vaccines based on the percentage of their population at risk of disease, and irrespective of their ability to place advance orders with vaccine manufacturers, or indeed, their ability to pay. In other words, equitable global access to vaccines in a pandemic is dependent on not only the *number* of doses accessible, but also the *timing* of access to vaccines.

**Figure 1:**
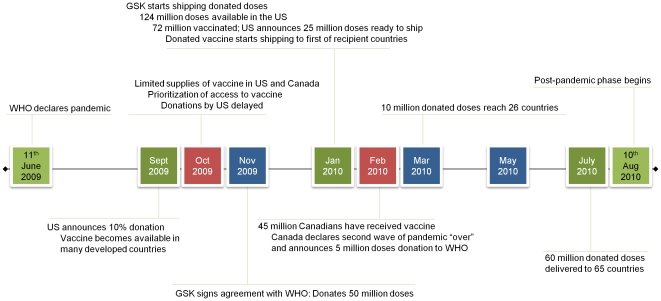
Timeline of the 2009 H1N1 Pandemic.

Based on these principles, early access to vaccines during the 2009 pandemic, which was largely dependent on nation states' ability to pay for advance purchase agreements, was inequitable. Many nations bought vaccine to cover a large proportion of their population. For example, Canada ordered 50 million doses for 33 million citizens and estimated that 45% of its population was immunized by January 2010 [5] (See Figure 1 for a timeline of vaccine availability and donations during the 2009 H1N1 pandemic). Though research in Canada showed that public support for increased global assistance—even at the expense of resources for Canadians—existed before the pandemic [6], it was only one day *after* the 2^nd^ wave of the flu pandemic was declared “over” in Canada that the country announced that it would donate five million excess vaccine doses to the WHO [7]. The US ordered ∼195 million doses at first, adding another 55 million to make a total of 250 million doses for a population of 300 million [8]. The country announced on September 17^th^ 2009 that it would donate 10% of its supply to poorer countries through the WHO [9]. However, when only limited vaccine became available in October 2009, vaccine was recommended for target groups in the US totaling about 160 million people [2] and vaccine donation was delayed until at-risk people in the US got the vaccine [10], [11]. By January 11^th^ 2010, vaccine shortages had eased considerably—61 million people in the US had received the vaccine—and US Secretary of Health and Human Services, Kathleen Sebelius, announced that 25 million doses were ready to ship to the WHO [12]. Only an estimated five million people accepted the vaccine in France, which bought 94 million doses; the country tried to sell excess vaccine to countries with no vaccine in January [13]. The irony associated with reduced uptake in developed countries of a vaccine that was completely unavailable to most of the world population has been commented on by others [14]. Manufacturers of the vaccine also donated doses. For example, GSK's vaccine was approved in September 2009 and the company pledged 50 million doses to the WHO in November 2009. It shipped these doses beginning in January 2010, approximately three months after vaccine was first shipped to paying customers [15].

Donations by manufacturers and nations to poorer countries were coordinated through the WHO. The WHO aimed to provide vaccine to 95 low- and middle-income countries, home to two billion people [16], to cover 10% of their population [3]. Two hundred million doses of vaccine were pledged to the WHO, including 120 million doses from US, Australia, Brazil, France, Italy, New Zealand, Norway, Switzerland, and Britain [17]. The first shipments of donated vaccine to arrive in a developing nation reached Mongolia in January 2010 [18] compared to the arrival and availability of vaccine in the US and France, for example, in October 2009 [2], [19]. As of August 2^nd^ 2010 (close to the official end of the pandemic), 65 countries had received 60.5 million donated vaccine doses [18].

In sum, sufficient vaccine was donated to cover 10% of the population of poor countries whereas much larger proportions of countries receiving the earliest doses were covered; the principle of equitable access, as defined by the Gates Foundation, was further violated because the timing of donations was much after those at risk in the donating nations had access to vaccine (See Figure 1 for a timeline of the pandemic).

Given a similar risk for specific populations across select nations—based on the hemisphere, season, number of detected cases, and demographic features of the population—we contend that it becomes a moral issue or an issue of justice for access to be dependent on a nation's ability to pay for doses through advance purchase agreements [20]. The nature of the 2009 H1N1 pandemic resulted in surplus doses in many countries easing the decision to donate purchased vaccine doses [21] and reduced demand from poorer nations; however, in the event of a more severe pandemic with higher mortality rates, demand could outstrip supply of vaccines, leaving nations unwilling to donate vaccine at all.

Facilitated by the WHO, the “Open-Ended Working Group of Member States” negotiated a “Pandemic Influenza Preparedness Framework for the Sharing of Influenza Viruses and Access to Vaccines and Other Benefits” [22], [23], [24]. The framework, presented to the World Health Assembly in 2011, authorizes the WHO to establish and maintain a stockpile of 150 million doses of H5N1 vaccine, of which 100 million doses are meant for distribution to developing countries in need once a pandemic begins [24]. Such a stockpile would enable timely access to vaccine in developing countries in the event of an H5N1 pandemic, though the adequacy of the size of the stockpile will depend on the severity of the pandemic. The framework also encourages WHO member states to “consider making donations and in-kind contributions” to the WHO to improve global pandemic preparedness. Efforts to build self-sufficiency in developing nations' vaccine production capabilities have been limited in the past and focused on diseases other than influenza [25]. The US recently announced grants to the WHO to strengthen developing countries' ability to produce flu vaccine [26], but voluntary donations by rich countries are likely to remain an important means of access to vaccines across the globe in an influenza pandemic in the foreseeable future in spite of the Pandemic Influenza Preparedness Framework [24], especially in the event of a severe pandemic, or a non-H5N1 pandemic.

Accurate knowledge of the potential public support for such a decision could be important to policy makers. We explored what the US public believes about vaccine donation by their country to poorer countries. We hypothesize that views on this issue will vary by demographic characteristics, at-risk status, party affiliation and political ideology, as well as whether the respondent has access to healthcare resources. We describe our findings and provide broad recommendations for researchers, advocacy groups, policy makers, and the public. We contend that on the one hand, governments of developed nations may be able to call on principles of fairness and equity to donate vaccine to poorer nations in a timely manner, and on the other, public and advocacy groups could, in turn, call upon those principles in advocating to policy makers for decisions to donate vaccine such that nations would have concurrent access to vaccines in a pandemic.

## Methods

This study was approved by the University of Pittsburgh Institutional Review Board. Knowledge Network provided an anonymized data file for analysis and all data were analyzed anonymously.

A representative sample of the US population was randomly drawn from the Knowledge Networks (KN) online research panel. To recruit people to the panel, KN uses random-digit dial and address-based probability sampling methods and provides panelists with access to the internet and hardware, if necessary. For this study, a national sample of 3689 adults aged ≥18, including oversamples of African American and Hispanics, was contacted by email. Between January 22^nd^ and February 1^st^ 2010, 2079 respondents completed the survey for a 56% completion rate. KN provided a data file with weighting variables, which incorporate design-based weights to account for the recruitment of panelists and both panel-based and study-specific post-stratification weights benchmarked against the most recent Current Population Survey (CPS) with respect to demographic and geographic distributions of the population aged ≥18. Information on the KN panel is available from their website [27],[28].

### Survey Instrument and Measures

The questionnaire focused on attitudes towards the 2009 H1N1 virus (identified interchangeably as swine flu) and donation of the H1N1 influenza vaccine. KN collects demographic variables as part of its research procedures.

To gauge access to healthcare resources in general, we asked if the respondent has health insurance. We assessed perceived access to the vaccine using our measure of subjective presence in a priority group for the vaccine. We measured “Subjective Priority” using the item, “Are you in a priority group to get the swine flu vaccine first?” (“No” and “Don't know” collapsed into “No”). At-risk status was assessed using “Objective Priority,” which was computed as a dichotomous variable based on the ACIP's recommendations [29]. We asked if respondents had been told by a health professional that they had any of 9 chronic medical conditions. We gave those aged 25–64 years with a chronic condition, pregnant women, respondents aged 18–24, and those who reported being caretakers of children aged <6 months or healthcare/emergency medical services personnel a score of 1; all others got a score of 0 (‘No’).

We asked two questions about influenza vaccine donation: “The US government is donating 10% of the 195 million doses of vaccine it has purchased to the World Health Organization (WHO) to distribute in poor countries that do not have the resources to buy their own vaccine.” We first asked how the respondent viewed willingness to donate the *amount* of vaccine being donated: “a. The US should not donate vaccine it has purchased; b. The US should donate less than 10% of its purchased vaccine; c. The US should donate more than 10% of its purchased amount of vaccine; d. A donation of 10% of its purchased vaccine by the US is just right.” Next, we asked how the respondent felt about the timing of the donation: “a. The US should donate vaccine after everyone who wants it here has gotten the vaccine; b. The US should donate vaccine after those at high-risk here have gotten the vaccine; c. The US should donate vaccine now so that people at risk in poor countries can get the vaccine at the same time as those at risk here.”

## Results

The demographic characteristics of the entire sample, including access to health insurance as well as subjective and objective priority, are shown in Table 1.

**Table 1:** Demographics of the overall sample.

	Overall	
*Characteristic*	N^a^	%^b^
*Total*	2079	100
*Income*		
Under 25 K	550	25.0
25 K–49 K	582	25.1
50 K–75 K	401	22.3
≥75 K	546	27.6
*Education*		
<High School	311	13.8
High School	674	30.8
Some College	591	28.1
≥Bachelor's degree	503	27.2
*Gender*		
Male	975	48.8
Female	1089	51.2
*Age, years*		
Mean (SE)	46.6 (0.70)	
*Race/Ethnicity*		
White, NH^c^	849	69.3
Black, NH^c^	591	11.6
Hispanic	602	14.2
Other, NH^c^	37	4.8
*Health Insurance*		
No	480	21.9
Yes	1565	78.1
*Subjective Priority*		
No	1661	81.9
Yes	399	18.1
*Objective Priority*		
No	1002	48.9
Yes	1077	51.1
*Born in US*		
No	398	11.9
Yes	1634	88.1
*Party*		
Republican	651	40.8
Independent	71	3.4
Democrat	1353	55.8
*Ideology*		
Liberal	626	29.8
Moderate	753	34.1
Conservative	681	36.1

a Unweighted N;

b Weighted %;

c Non-Hispanic.

### Views on the amount of vaccine donated

As shown in Table 2, 55.7% of respondents felt that a donation of 10% of purchased vaccine by the US to poorer countries was “just right.” Greater than 21% felt the US should donate more than 10% of its vaccine, in contrast to only 16% who felt that the country should not donate vaccine at all, and 6.8% who felt that a donation of less than 10% would be appropriate.

**Table 2:** Characteristics of respondents to question regarding the scale of vaccine donation.

		N (%)†				
US is donating 10% of its purchased vaccine; How do you feel about the amount?*		Should not donate vaccine	Should donate <10%	Should donate >10%	A donation of 10% is just right	p-value
**Total (N = 1999)**		237 (16.0)	122 (6.8)	539 (21.5)	1101 (55.7)	
**Income**	<$25 K	36 (8.2)	33 (6.9)	170 (27.9)	279 (57.0)	0.014
	$25–49 K	63 (20.9)	25 (4.0)	164 (19.3)	314 (55.8)	
	$50–74 K	60 (18.2)	27 (8.6)	89 (20.5)	212 (52.7)	
	≥$75 K	78 (16.6)	37 (7.8)	116 (18.7)	296 (57.0)	
**Education**	<High School	22 (12.0)	20 (7.0)	94 (22.3)	154 (58.7)	0.275
	High School	68 (16.0)	37 (8.3)	185 (21.2)	354 (54.4)	
	Some College	86 (22.9)	34 (6.5)	135 (21.2)	319 (49.4)	
	≥Bachelor's Degree	61 (10.7)	31 (5.3)	125 (21.8)	274 (62.2)	
**Health Insurance**	No	44 (12.6)	26 (5.2)	154 (23.0)	243 (59.2)	0.352
	Yes	189 (16.8)	96 (7.3)	379 (21.0)	855 (54.8)	
**Subjective Priority**	No	193 (17.0)	100 (6.3)	427 (21.0)	878 (55.7)	0.297
	Yes	43 (11.7)	22 (9.1)	109 (23.8)	221 (55.4)	
**Objective Priority**	No	115 (17.4)	62 (8.6)	227 (16.5)	559 (57.4)	0.005
	Yes	122 (14.6)	60 (5.1)	312 (26.4)	542 (54.0)	
**Born in US**	No	21 (8.8)	15 (5.9)	157 (36.9)	191 (48.3)	<0.001
	Yes	209 (16.7)	106 (7.2)	371 (19.4)	892 (56.8)	
**Party**	Republican	130 (22.0)	47 (6.0)	97 (14.3)	354 (57.6)	<0.001
	Democrat	103 (11.4)	74 (7.7)	420 (26.8)	715 (54.1)	
**Ideology**	Liberal	47 (12.1)	34 (6.6)	209 (28.8)	314 (52.4)	<0.001
	Moderate	81 (15.4)	35 (5.7)	199 (24.4)	412 (54.5)	
	Conservative	109 (19.9)	52 (7.9)	127 (12.7)	369 (59.5)	

† Unweighted N, Weighted %;

* See Methods for exact wording.

Income (p = 0.014), objective priority status (p = 0.005), nativity, party affiliation, and political ideology (p<0.001) were significantly related to views on the amount of vaccine to be donated (Table 2). Close to 28% of people in the lowest income category believed that the US should donate greater than 10% of purchased vaccine compared to close to 20% of those in higher income categories. Whereas subjective priority was not significantly related to views on vaccine donation, 26.4% of those objectively in a priority group to receive vaccine compared to 16.5% of those not in a priority group to receive vaccine felt that the US should donate more than 10% of its purchased vaccine. Nativity (born in the US or not) was significantly related to views on the amount of vaccine donation (p<0.001). These results suggest that immigrants, poorer people, and those at greater risk in the US were more willing to share vaccine than others were.

We found that party affiliation and political ideology were related to vaccine donation (p<0.001). According to our study, greater than 26% of Democrats felt that the country should donate greater than 10% of its purchased vaccine compared to 14.3% of Republicans. Yet, greater than 50% of each believed that a donation of 10% of vaccine by the US was “just right.” Similarly, 28.8% of liberals but only 12.7% of conservatives believed that the US should donate more than 10% of its vaccine; a majority of conservatives, moderates, and liberals, however, believed that a donation of 10% was “just right” (Table 2). We obtained similar results in a multinomial logistic regression model examining views on the amount of vaccine donated as the outcome and the characteristics shown in Table 2 as independent variables though ideology was no longer related to the outcome after controlling for party affiliation (results available on request).

### Views on the timing of donations

All respondents other than those who felt that the US should not donate any vaccine were asked if vaccine should be donated after those in the US had received it or at a time such that those at risk in other countries could get it concurrently with those at risk in the US. 44.9% felt that vaccine should be donated “now so that people at risk in poor countries can get the vaccine at the same time as those at risk here” compared to 31.3% who felt that donations should be made after those at risk here got the vaccine and 23.8% who felt that donations should happen only after those who *want* the vaccine in the US had received it (Table 3).

**Table 3:** Characteristics of respondents to question regarding the timing of vaccine donation.

		N (%)†			
US is donating 10% of its purchased vaccine; How do you feel about the timing?*		Donate after those who want get it*	Donate after at-risk get it*	Donate now so that at-risk everywhere have access*	p-value
**Total (N = 1731)**		395 (23.8)	455 (31.3)	881 (44.9)	
**Income**	<$25 K	98 (23.5)	98 (26.7)	274 (49.7)	0.129
	$25–49 K	124 (27.4)	120 (26.6)	255 (46.0)	
	$50–74 K	69 (17.2)	98 (37.1)	158 (45.7)	
	≥$75 K	104 (26.4)	139 (35.1)	194 (38.5)	
**Education**	<High School	50 (17.3)	58 (24.5)	153 (58.2)	0.030
	High School	140 (31.4)	124 (26.4)	303 (42.3)	
	Some College	111 (19.2)	150 (40.0)	219 (40.8)	
	≥Bachelor's Degree	94 (23.1)	123 (32.0)	206 (44.9)	
**Health Insurance**	No	95 (23.2)	98 (24.9)	223 (51.9)	0.166
	Yes	300 (24.1)	356 (33.3)	651 (42.6)	
**Subjective Priority**	No	315 (23.0)	369 (32.0)	697 (45.0)	0.675
	Yes	79 (27.3)	86 (28.9)	180 (43.8)	
**Objective Priority**	No	186 (20.9)	215 (31.3)	437 (47.8)	0.236
	Yes	209 (26.6)	240 (31.3)	444 (42.1)	
**Born in US**	No	58 (17.4)	64 (24.5)	234 (58.1)	0.001
	Yes	329 (24.7)	385 (33.2)	631 (42.1)	
**Party**	Republican	146 (28.1)	134 (32.5)	212 (39.4)	0.100
	Democrat	239 (20.9)	311 (30.8)	637 (48.3)	
**Ideology**	Liberal	104 (19.0)	150 (28.3)	290 (52.7)	0.090
	Moderate	145 (22.1)	171 (35.8)	316 (42.1)	
	Conservative	143 (29.8)	133 (29.6)	268 (40.6)	

† Unweighted N, Weighted %;

* See Methods for exact wording.

Education (p = 0.030) and nativity (p = 0.001) were related to views on the timing of donations. Greater than 58% of those with the lowest level of education but less than 45% of those with higher levels of education felt that donations should occur so that those at risk in poorer countries have access to the vaccine at the same time as those at risk here. Of those born outside the US, 58.1%, whereas 42.1% of those born in the US believed that donations should enable access to those at risk in poorer countries at the same time as those at risk in the US. Somewhat surprisingly, other demographic and party affiliation/ideological characteristics were not significantly related to views on the *timing* of vaccine donations. Though education remained significantly related to views on the timing of donations in a multinomial logistic regression model with all characteristics in Table 3 as independent variables, nativity was no longer significantly related to the outcome (results available on request).

### Support for prioritization in determining who gets limited supplies of vaccine in the US

Donating larger amounts of purchased vaccine or donating vaccine early such that those at risk elsewhere have access concurrently with those at risk in the US would necessitate prioritization in the US to determine who gets limited supplies of vaccine. We examined support for such prioritization. As shown in Table 4, bipartisan support for such prioritization in the US exists (p = 0.394 for a difference between Republicans and Democrats), providing evidence that larger and earlier donations leading to limited availability and prioritization of vaccine in the US may be politically possible.

**Table 4:** Bipartisan support for prioritization in determining who gets limited supplies of vaccines or drugs.

		Setting priorities on limited supplies of vaccine N (%)*				p-value
		Strongly Oppose	Oppose	Favor	Strongly Favor	
**Total**		219 (10.3)	551 (24.9)	1019 (55.5)	214 (9.3)	
**Party**	Republican	74 (11.9)	168 (23.3)	326 (54.4)	64 (10.4)	0.394
	Democrat	136 (8.4)	365 (25.7)	667 (57.3)	144 (8.6)	

* Unweighted N, Weighted %.

## Discussion

Ensuring timely access to influenza vaccine throughout the world is a public health goal of the WHO. We have shown that there exists considerable support for timely donations of at least 10% of the US share of vaccines during a pandemic. We acknowledge, however, that our study was conducted in the context of a pandemic in which illness was mild for most and the overall fatality rate was lower than the severe 1918 Spanish influenza pandemic with which it was often compared in media characterizations of the pandemic. The perception that the disease was mild may have played a role in recipients' support for timely vaccine donations. Our results encourage further research—including experimental studies employing hypothetical scenarios such as pandemics caused by a virulent viral strain—to understand the public's potential attitudes toward vaccine donations in a severe pandemic.

Vaccine donations are a political and fiscal decision within donor countries. Developing political consensus for timely donation of influenza vaccines *during* a pandemic is a challenge. For example, though research showed that public support for increased global assistance during a pandemic existed in Canada [6], the absence of public and political discourse regarding this issue presumably made it impossible for the government to donate excess doses until after the 2^nd^ wave of the flu pandemic was declared “over” [7]. It may also be challenging to garner the fiscal resources to support vaccine availability internally as well as to ensure adequate supply for donation to other countries. Therefore, to address these policy decisions requires discussion and planning *before* as opposed to *during* the next pandemic. This may be increasingly important in the current fiscal environment for many countries.

Our results suggest that there is more public support for vaccine donations in the US than policy makers may have expected. Considerable bi-partisan—though short of majority—support exists for the *timely* donation of 10% of vaccine. If global health advocacy groups wanted to boost support within the US for vaccine donations to poorer countries, they could potentially frame either a moral argument that is based on justice or a utilitarian argument that we live in a global economy, which makes it critically important for economic reasons that vaccine must be available throughout the world in order to maintain the ‘just-in-time’ distribution of products and goods across the globe. Arguments can also be centered on maintaining basic capabilities and functionings of people in poorer countries [30]: in countries that lack adequate access to high quality *curative* healthcare, increased access to vaccines (at a level depending on people's vulnerability) could be imperative if people are to maintain their livelihood and other capabilities during a pandemic. Arguments could be received differently, however, depending on the political ideology of the recipient. Recent multi-stage research conducted recently—employing qualitative focus groups and quantitative survey methods—furthered understanding of not only how the public and opinion elites understand messages dealing with another public health issue with moral underpinnings—health disparities and the social determinants of health—but also of wording of messages that would most resonate with the public and build support for evidence-based policy change [31]. We suggest the need for similar research on message wording that would resonate in a bi-partisan manner to build further support for vaccine donations in future pandemics.

We found support for donation among lowest income groups who were, in fact, the most likely to support donation of more than 10%. That nativity was related to views on the amount and timing of vaccine donations is a potentially interesting finding. In the absence, however, of knowledge regarding the country of origin for those not born in the US, we hesitate to draw a generalizable conclusion from this finding.

Although we found substantial support for donation, the 2009 H1N1 pandemic was perceived by many to be mild and it is unclear what the impact on attitudes would be if mortality rates were higher in a pandemic. Future research should explore the extent to which severity may affect public attitudes and political calculations of policy makers. While one might speculate that severity may decrease public willingness to support donation, an economic and pragmatic argument, which focuses on the critical importance of vaccine worldwide to maintain the global just in time economy, may yet be persuasive. Policy makers may also imagine that in a more severe pandemic, support for donation would decrease if it requires prioritization of vaccine distribution in the US. However, we did find bipartisan support for prioritization of vaccine recipients; prioritization would, in reality, *have* to occur, at least initially in a pandemic, regardless of vaccine donation decisions.

Ensuring that influenza vaccine is available in adequate supplies and distributed in a timely manner during an influenza pandemic is a global public health goal. As part of the ‘Open-Ended Working Group of Member States’ of the WHO [22], countries negotiated a potential material transfer agreement that will govern the transfer and use of biological materials and resulting products during a pandemic. Neither the agreement nor our study addresses the issue of delays in vaccine delivery *after* receipt of donated shipments by the WHO or the ethical implications of requiring poorer nations to meet stringent conditions before receiving donation shipments; these are subjects for future research.

The new ‘Open-Ended Working Group of Member States’ framework [24] stipulates that a recipient (such as a vaccine manufacturer) of viral material commit to donating “at least 10% of real-time pandemic vaccine production to the WHO,” and reserve an equal amount “at affordable prices to WHO.” Faced with a severe pandemic, however, demand for scarce vaccine in poorer nations could outstrip the 20% of production available to the WHO under this framework. In such a scenario, it may become morally imperative for countries with large advance purchase agreements with vaccine manufacturers to consider donating vaccine to poorer countries. We argue that in light of our results, it is important for US policy makers as well as international partners to have a clear understanding of existing public support for timely vaccine donations. In order to frame arguments that would resonate in a bi-partisan manner, further research on communication strategies is required. The results of such research could inform methods to bolster existing support among the public and to advocate with policy makers on this issue prior to the next pandemic.
